# 2-[4-(2-Hy­droxy­eth­oxy)phenyl]-4,4,5,5-tetra­methyl-2-imidazoline-1-oxyl 3-oxide

**DOI:** 10.1107/S160053681104815X

**Published:** 2011-11-19

**Authors:** Lin-Lin Jing, Hui-Ping Ma, Xiao-Fei Fan, Lei He, Zheng-Ping Jia

**Affiliations:** a Department of Pharmacy, Lanzhou General Hospital of PLA, Key Laboratory of the Prevention and Cure of Plateau Environmental Damage, PLA 730050, Lanzhou Gansu, People’s Republic of China

## Abstract

In the title compound, C_15_H_21_N_2_O_4_, the imidazoline ring displays a twisted conformation. The dihedral angle between the mean plane of the imidazoline ring and the benzene ring is 33.50 (12)°. In the crystal, mol­ecules are connected by O—H⋯O hydrogen bonds, forming a zigzag chain along the *c* axis. The chains are linked by C—H⋯O and C—H⋯π inter­actions.

## Related literature

For the preparation of the title compound, see: Ullman *et al.* (1974 ▶). For biological properties of nitronyl nitroxides, see: Soule *et al.* (2007 ▶); Blasig *et al.* (2002 ▶); Qin *et al.* (2009 ▶); Tanaka *et al.* (2007 ▶). For coordination properties of nitronyl nitroxides, see: Masuda *et al.* (2009 ▶). For related structures, see: Wang *et al.* (2009 ▶); Jing *et al.* (2009 ▶). For puckering parameters, see: Cremer & Pople (1975 ▶). For pseudorotation parameters, see: Rao *et al.* (1981 ▶).
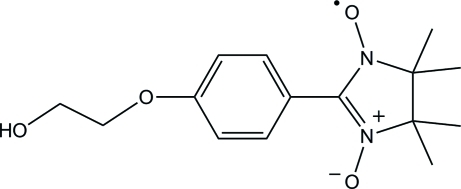


## Experimental

### 

#### Crystal data

C_15_H_21_N_2_O_4_
*M* *_r_* = 293.34
Orthorhombic, 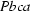

*a* = 8.869 (3) Å
*b* = 16.050 (5) Å
*c* = 20.925 (6) Å
*V* = 2978.7 (16) Å^3^
*Z* = 8
Mo *K*α radiation
μ = 0.10 mm^−1^
*T* = 296 K
0.26 × 0.23 × 0.22 mm

#### Data collection

Bruker APEXII CCD diffractometer
Absorption correction: multi-scan (*SADABS*; Bruker, 2007 ▶) *T*_min_ = 0.976, *T*_max_ = 0.979
20164 measured reflections
2774 independent reflections
1928 reflections with *I* > 2σ(*I*)
*R*_int_ = 0.054

#### Refinement

*R*[*F* ^2^ > 2σ(*F* ^2^)] = 0.047
*wR*(*F* ^2^) = 0.163
*S* = 0.95
2774 reflections
195 parameters
H-atom parameters constrained
Δρ_max_ = 0.22 e Å^−3^
Δρ_min_ = −0.25 e Å^−3^

### 

Data collection: *APEX2* (Bruker, 2007 ▶); cell refinement: *SAINT* (Bruker, 2007 ▶); data reduction: *SAINT*; program(s) used to solve structure: *SHELXS97* (Sheldrick, 2008 ▶); program(s) used to refine structure: *SHELXL97* (Sheldrick, 2008 ▶); molecular graphics: *ORTEP-3* (Farrugia, 1997 ▶); software used to prepare material for publication: *SHELXTL* (Sheldrick, 2008 ▶) and *PLATON* (Spek, 2009 ▶).

## Supplementary Material

Crystal structure: contains datablock(s) I, global. DOI: 10.1107/S160053681104815X/is5006sup1.cif

Structure factors: contains datablock(s) I. DOI: 10.1107/S160053681104815X/is5006Isup2.hkl

Additional supplementary materials:  crystallographic information; 3D view; checkCIF report

## Figures and Tables

**Table 1:** Hydrogen-bond geometry (Å, °) *Cg*2 is the centroid of the benzene C4–C9 ring.

*D*—H⋯*A*	*D*—H	H⋯*A*	*D*⋯*A*	*D*—H⋯*A*
O4—H4⋯O2^i^	0.82	2.01	2.828 (3)	173
C12—H12*C*⋯O1^ii^	0.96	2.54	3.418 (3)	152
C15—H15*C*⋯*Cg*2^iii^	0.96	2.80	3.570 (3)	138

Symmetry codes: (i) 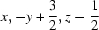; (ii) 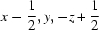; (iii) 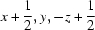.

**Fig. 1.**
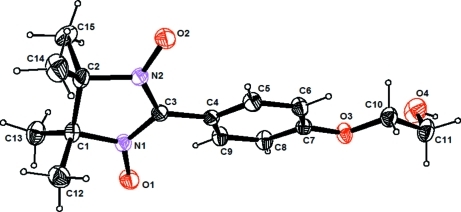
Molecular structure of the title compound, showing the atom labeling scheme. Displacement ellipsoids are drawn at the 30% probability level.

## supplementary crystallographic information

### Comment

Nitronyl nitroxides, firstly synthesized more than 30 years ago, can be used for coordination with many metal cations, such as Mn^2+^, Cu^2+^ and Ni^2+^ leading to form some molecule-based magnetic materials (Masuda *et al.*, 2009). They can also react with free radicals such as OH, H_2_O_2_ and O_2_ (Blasig *et al.*, 2002) to protect cells from the attack of free radicals. So they have a lot of biological properties as anticancer, antiradiation and antioxidation (Qin *et al.*, 2009; Tanaka *et al.*, 2007; Soule *et al.*, 2007).

The molecular structure of the title compound is shown in Fig. 1. The least-squares plane of the nitronyl nitroxide ring and the benzene ring are twisted with respect to each other making a dihedral angle of 33.50 (12)°. The puckering parameters of the nitronyl nitroxide ring are Q(2) = 0.177 (2) Å and φ = 237.1 (7)° (Cremer & Pople, 1975). The pseudorotation parameters (Rao *et al.*, 1981) for the nitronyl nitroxide ring are P = 39.7 (4)° and τ(*M*) = 18.2 (1) ° for the C1—N1 reference bond with the closest puckering descriptor being twisted on C1—C2. The crystal structure is stabilized by O—H···O, C—H···O and C—H···π hydrogen bonds (Table 1).

### Experimental

2,3-Dimethyl-2,3-bis(hydroxylamino) butane (1.48 g, 10.0 mmol) and 4-(4-hydroxyethoxy)benzaldehyde (1.66 g, 10 mmol) were dissolved in methanol (30.0 ml). The reaction was filtered after stirring for 24 h at room temperature. The resulting white powder was washed by cool methanol and suspended in the solution of dichloromethane (30.0 ml). Then the reaction mixture was added to an aqueous solution of NaIO_4_(30 ml) and stirred for 15 min in an ice bath to give a dark blue solution. The aqueous phase was extracted with CH_2_Cl_2_ and the organic layer was combined and dried over Na_2_SO_4_. Then the solvent was removed to give a dark blue residue which was purified by flash column chromatography with the elution of *n*-hexane/ ethyl acetate (1:2) to yield 1.61 g (55%) of the title compound as a dark blue powder. Single crystals of the title compound suitable for X-ray diffraction was recrystallized from hexane/dichloromethane (1:1).

### Refinement

All H atoms were positioned geometrically and refined using a riding model, with C—H = 0.96 Å (methyl), 0.97 Å (methylene) or 0.93 Å (aryl), and O—H = 0.82 Å, and with *U*_iso_(H) = 1.2*U*_eq_(C) or 1.5*U*_eq_(methyl C).

### Crystal data

**Table:**

C_15_H_21_N_2_O_4_	*F*(000) = 1256
*M**_r_* = 293.34	*D*_x_ = 1.308 Mg m^−^^3^
Orthorhombic, *P**b**c**a*	Mo *K*α radiation, λ = 0.71073 Å
Hall symbol: -P 2ac 2ab	Cell parameters from 3005 reflections
*a* = 8.869 (3) Å	θ = 2.5–21.6°
*b* = 16.050 (5) Å	µ = 0.10 mm^−^^1^
*c* = 20.925 (6) Å	*T* = 296 K
*V* = 2978.7 (16) Å^3^	Block, blue
*Z* = 8	0.26 × 0.23 × 0.22 mm

### Data collection

**Table:**

Bruker APEXII CCD diffractometer	2774 independent reflections
Radiation source: fine-focus sealed tube	1928 reflections with *I* > 2σ(*I*)
graphite	*R*_int_ = 0.054
φ and ω scans	θ_max_ = 25.5°, θ_min_ = 2.5°
Absorption correction: multi-scan (*SADABS*; Bruker, 2007)	*h* = −10→10
*T*_min_ = 0.976, *T*_max_ = 0.979	*k* = −17→19
20164 measured reflections	*l* = −25→25

### Refinement

**Table:**

Refinement on *F*^2^	Primary atom site location: structure-invariant direct methods
Least-squares matrix: full	Secondary atom site location: difference Fourier map
*R*[*F*^2^ > 2σ(*F*^2^)] = 0.047	Hydrogen site location: inferred from neighbouring sites
*wR*(*F*^2^) = 0.163	H-atom parameters constrained
*S* = 0.95	*w* = 1/[σ^2^(*F*_o_^2^) + (0.1*P*)^2^ + 0.8575*P*] where *P* = (*F*_o_^2^ + 2*F*_c_^2^)/3
2774 reflections	(Δ/σ)_max_ < 0.001
195 parameters	Δρ_max_ = 0.22 e Å^−^^3^
0 restraints	Δρ_min_ = −0.25 e Å^−^^3^

### Special details

**Table:**

Geometry. All e.s.d.'s (except the e.s.d. in the dihedral angle between two l.s. planes) are estimated using the full covariance matrix. The cell e.s.d.'s are taken into account individually in the estimation of e.s.d.'s in distances, angles and torsion angles; correlations between e.s.d.'s in cell parameters are only used when they are defined by crystal symmetry. An approximate (isotropic) treatment of cell e.s.d.'s is used for estimating e.s.d.'s involving l.s. planes.
Refinement. Refinement of *F*^2^ against ALL reflections. The weighted *R*-factor *wR* and goodness of fit *S* are based on *F*^2^, conventional *R*-factors *R* are based on *F*, with *F* set to zero for negative *F*^2^. The threshold expression of *F*^2^ > σ(*F*^2^) is used only for calculating *R*-factors(gt) *etc*. and is not relevant to the choice of reflections for refinement. *R*-factors based on *F*^2^ are statistically about twice as large as those based on *F*, and *R*- factors based on ALL data will be even larger.

### Fractional atomic coordinates and isotropic or equivalent isotropic displacement parameters (Å^2^)

**Table:**

	*x*	*y*	*z*	*U*_iso_*/*U*_eq_	
C1	0.8584 (2)	1.12763 (14)	0.33524 (10)	0.0381 (5)	
C2	0.8507 (3)	1.05283 (14)	0.38244 (10)	0.0401 (5)	
C3	0.8131 (2)	1.00297 (13)	0.27718 (9)	0.0338 (5)	
C4	0.7901 (2)	0.94682 (13)	0.22347 (9)	0.0333 (5)	
C5	0.6890 (3)	0.88085 (13)	0.22601 (10)	0.0405 (5)	
H5	0.6343	0.8719	0.2633	0.049*	
C6	0.6678 (3)	0.82848 (13)	0.17472 (10)	0.0405 (5)	
H6	0.5986	0.7851	0.1773	0.049*	
C7	0.7503 (3)	0.84085 (13)	0.11912 (9)	0.0351 (5)	
C8	0.8535 (3)	0.90560 (15)	0.11621 (10)	0.0417 (6)	
H8	0.9098	0.9136	0.0792	0.050*	
C9	0.8733 (3)	0.95792 (14)	0.16734 (10)	0.0399 (6)	
H9	0.9427	1.0012	0.1647	0.048*	
C10	0.6427 (3)	0.72306 (15)	0.06578 (10)	0.0463 (6)	
H10A	0.5408	0.7395	0.0770	0.056*	
H10B	0.6778	0.6829	0.0971	0.056*	
C11	0.6449 (3)	0.68517 (19)	0.00040 (12)	0.0586 (7)	
H11A	0.5780	0.6374	−0.0007	0.070*	
H11B	0.6087	0.7255	−0.0305	0.070*	
C12	0.7205 (3)	1.18423 (16)	0.33598 (13)	0.0550 (7)	
H12A	0.7275	1.2236	0.3016	0.083*	
H12B	0.7161	1.2135	0.3759	0.083*	
H12C	0.6311	1.1512	0.3309	0.083*	
C13	1.0013 (3)	1.17980 (17)	0.33876 (13)	0.0569 (7)	
H13A	1.0871	1.1452	0.3300	0.085*	
H13B	1.0106	1.2034	0.3807	0.085*	
H13C	0.9962	1.2238	0.3077	0.085*	
C14	0.7400 (4)	1.06293 (18)	0.43721 (12)	0.0653 (8)	
H14A	0.6413	1.0741	0.4204	0.098*	
H14B	0.7711	1.1085	0.4638	0.098*	
H14C	0.7375	1.0126	0.4620	0.098*	
C15	1.0041 (3)	1.02474 (19)	0.40759 (13)	0.0638 (8)	
H15A	0.9929	0.9733	0.4305	0.096*	
H15B	1.0440	1.0665	0.4357	0.096*	
H15C	1.0720	1.0167	0.3724	0.096*	
N1	0.8557 (2)	1.08321 (11)	0.27221 (8)	0.0368 (4)	
N2	0.7976 (2)	0.98429 (11)	0.33930 (8)	0.0374 (5)	
O1	0.8756 (2)	1.12342 (10)	0.21999 (7)	0.0558 (5)	
O2	0.7595 (2)	0.91285 (10)	0.36180 (7)	0.0581 (5)	
O3	0.73915 (18)	0.79412 (10)	0.06528 (7)	0.0464 (4)	
O4	0.7906 (3)	0.66024 (15)	−0.01595 (10)	0.0783 (7)	
H4	0.7890	0.6369	−0.0508	0.117*	

### Atomic displacement parameters (Å^2^)

**Table:**

	*U*^11^	*U*^22^	*U*^33^	*U*^12^	*U*^13^	*U*^23^
C1	0.0401 (12)	0.0392 (13)	0.0350 (11)	−0.0033 (10)	0.0006 (9)	−0.0095 (9)
C2	0.0493 (13)	0.0421 (13)	0.0288 (10)	−0.0015 (10)	−0.0004 (9)	−0.0067 (9)
C3	0.0397 (11)	0.0330 (12)	0.0289 (10)	0.0003 (10)	0.0010 (8)	0.0005 (8)
C4	0.0399 (12)	0.0319 (11)	0.0280 (10)	0.0023 (9)	−0.0011 (8)	0.0003 (8)
C5	0.0521 (13)	0.0402 (13)	0.0293 (10)	−0.0037 (11)	0.0089 (9)	−0.0004 (9)
C6	0.0537 (14)	0.0347 (12)	0.0331 (11)	−0.0076 (10)	0.0056 (10)	−0.0016 (9)
C7	0.0421 (12)	0.0342 (11)	0.0289 (10)	0.0037 (9)	−0.0009 (9)	−0.0045 (8)
C8	0.0444 (12)	0.0491 (14)	0.0315 (11)	−0.0040 (11)	0.0113 (9)	−0.0042 (9)
C9	0.0422 (13)	0.0423 (13)	0.0352 (11)	−0.0078 (10)	0.0065 (9)	−0.0050 (9)
C10	0.0536 (14)	0.0489 (14)	0.0363 (11)	−0.0112 (11)	−0.0006 (10)	−0.0037 (10)
C11	0.0661 (18)	0.0676 (18)	0.0420 (14)	−0.0164 (14)	−0.0063 (12)	−0.0132 (12)
C12	0.0545 (16)	0.0485 (15)	0.0621 (16)	0.0082 (12)	−0.0003 (12)	−0.0064 (12)
C13	0.0537 (16)	0.0619 (17)	0.0550 (15)	−0.0171 (13)	−0.0008 (12)	−0.0081 (13)
C14	0.092 (2)	0.0601 (17)	0.0438 (14)	−0.0058 (15)	0.0248 (14)	−0.0106 (12)
C15	0.0679 (18)	0.0708 (19)	0.0527 (15)	0.0060 (15)	−0.0233 (14)	0.0018 (13)
N1	0.0449 (11)	0.0363 (10)	0.0293 (9)	−0.0035 (8)	0.0014 (7)	−0.0010 (7)
N2	0.0505 (11)	0.0355 (10)	0.0261 (8)	−0.0023 (8)	0.0013 (7)	0.0005 (7)
O1	0.0892 (14)	0.0420 (10)	0.0363 (9)	−0.0110 (9)	0.0059 (8)	0.0083 (7)
O2	0.0993 (14)	0.0428 (10)	0.0322 (8)	−0.0155 (10)	0.0016 (8)	0.0072 (7)
O3	0.0640 (11)	0.0435 (9)	0.0317 (8)	−0.0112 (8)	0.0074 (7)	−0.0106 (6)
O4	0.0952 (17)	0.0864 (16)	0.0532 (12)	0.0100 (13)	0.0002 (10)	−0.0280 (10)

### Geometric parameters (Å, °)

**Table:**

C1—N1	1.499 (3)	C10—C11	1.497 (3)
C1—C13	1.520 (3)	C10—H10A	0.9700
C1—C12	1.524 (3)	C10—H10B	0.9700
C1—C2	1.556 (3)	C11—O4	1.395 (3)
C2—N2	1.499 (3)	C11—H11A	0.9700
C2—C14	1.518 (3)	C11—H11B	0.9700
C2—C15	1.527 (4)	C12—H12A	0.9600
C3—N2	1.341 (3)	C12—H12B	0.9600
C3—N1	1.346 (3)	C12—H12C	0.9600
C3—C4	1.455 (3)	C13—H13A	0.9600
C4—C5	1.389 (3)	C13—H13B	0.9600
C4—C9	1.398 (3)	C13—H13C	0.9600
C5—C6	1.376 (3)	C14—H14A	0.9600
C5—H5	0.9300	C14—H14B	0.9600
C6—C7	1.389 (3)	C14—H14C	0.9600
C6—H6	0.9300	C15—H15A	0.9600
C7—O3	1.357 (2)	C15—H15B	0.9600
C7—C8	1.386 (3)	C15—H15C	0.9600
C8—C9	1.371 (3)	N1—O1	1.281 (2)
C8—H8	0.9300	N2—O2	1.285 (2)
C9—H9	0.9300	O4—H4	0.8200
C10—O3	1.426 (3)		
			
N1—C1—C13	108.57 (17)	O4—C11—C10	110.7 (2)
N1—C1—C12	106.21 (18)	O4—C11—H11A	109.5
C13—C1—C12	109.9 (2)	C10—C11—H11A	109.5
N1—C1—C2	101.00 (16)	O4—C11—H11B	109.5
C13—C1—C2	115.53 (19)	C10—C11—H11B	109.5
C12—C1—C2	114.71 (19)	H11A—C11—H11B	108.1
N2—C2—C14	109.3 (2)	C1—C12—H12A	109.5
N2—C2—C15	105.71 (19)	C1—C12—H12B	109.5
C14—C2—C15	110.4 (2)	H12A—C12—H12B	109.5
N2—C2—C1	101.41 (16)	C1—C12—H12C	109.5
C14—C2—C1	115.2 (2)	H12A—C12—H12C	109.5
C15—C2—C1	114.0 (2)	H12B—C12—H12C	109.5
N2—C3—N1	108.50 (17)	C1—C13—H13A	109.5
N2—C3—C4	126.58 (19)	C1—C13—H13B	109.5
N1—C3—C4	124.91 (18)	H13A—C13—H13B	109.5
C5—C4—C9	118.04 (18)	C1—C13—H13C	109.5
C5—C4—C3	122.21 (18)	H13A—C13—H13C	109.5
C9—C4—C3	119.75 (19)	H13B—C13—H13C	109.5
C6—C5—C4	121.61 (19)	C2—C14—H14A	109.5
C6—C5—H5	119.2	C2—C14—H14B	109.5
C4—C5—H5	119.2	H14A—C14—H14B	109.5
C5—C6—C7	119.6 (2)	C2—C14—H14C	109.5
C5—C6—H6	120.2	H14A—C14—H14C	109.5
C7—C6—H6	120.2	H14B—C14—H14C	109.5
O3—C7—C8	115.21 (18)	C2—C15—H15A	109.5
O3—C7—C6	125.3 (2)	C2—C15—H15B	109.5
C8—C7—C6	119.47 (18)	H15A—C15—H15B	109.5
C9—C8—C7	120.63 (19)	C2—C15—H15C	109.5
C9—C8—H8	119.7	H15A—C15—H15C	109.5
C7—C8—H8	119.7	H15B—C15—H15C	109.5
C8—C9—C4	120.6 (2)	O1—N1—C3	125.90 (17)
C8—C9—H9	119.7	O1—N1—C1	120.55 (17)
C4—C9—H9	119.7	C3—N1—C1	113.06 (16)
O3—C10—C11	108.08 (19)	O2—N2—C3	125.57 (17)
O3—C10—H10A	110.1	O2—N2—C2	121.12 (16)
C11—C10—H10A	110.1	C3—N2—C2	112.80 (17)
O3—C10—H10B	110.1	C7—O3—C10	118.67 (16)
C11—C10—H10B	110.1	C11—O4—H4	109.5
H10A—C10—H10B	108.4		
			
N1—C1—C2—N2	−16.7 (2)	N2—C3—N1—O1	−177.2 (2)
C13—C1—C2—N2	−133.60 (19)	C4—C3—N1—O1	3.5 (3)
C12—C1—C2—N2	97.0 (2)	N2—C3—N1—C1	−5.2 (2)
N1—C1—C2—C14	−134.5 (2)	C4—C3—N1—C1	175.45 (19)
C13—C1—C2—C14	108.6 (2)	C13—C1—N1—O1	−51.1 (3)
C12—C1—C2—C14	−20.8 (3)	C12—C1—N1—O1	67.0 (2)
N1—C1—C2—C15	96.4 (2)	C2—C1—N1—O1	−172.97 (19)
C13—C1—C2—C15	−20.5 (3)	C13—C1—N1—C3	136.5 (2)
C12—C1—C2—C15	−149.9 (2)	C12—C1—N1—C3	−105.4 (2)
N2—C3—C4—C5	29.5 (3)	C2—C1—N1—C3	14.6 (2)
N1—C3—C4—C5	−151.3 (2)	N1—C3—N2—O2	−179.3 (2)
N2—C3—C4—C9	−149.5 (2)	C4—C3—N2—O2	0.0 (4)
N1—C3—C4—C9	29.7 (3)	N1—C3—N2—C2	−7.4 (2)
C9—C4—C5—C6	−1.3 (3)	C4—C3—N2—C2	171.86 (19)
C3—C4—C5—C6	179.6 (2)	C14—C2—N2—O2	−49.8 (3)
C4—C5—C6—C7	0.7 (4)	C15—C2—N2—O2	69.0 (3)
C5—C6—C7—O3	−179.5 (2)	C1—C2—N2—O2	−171.79 (19)
C5—C6—C7—C8	0.3 (3)	C14—C2—N2—C3	138.0 (2)
O3—C7—C8—C9	179.1 (2)	C15—C2—N2—C3	−103.3 (2)
C6—C7—C8—C9	−0.8 (3)	C1—C2—N2—C3	15.9 (2)
C7—C8—C9—C4	0.2 (3)	C8—C7—O3—C10	176.0 (2)
C5—C4—C9—C8	0.8 (3)	C6—C7—O3—C10	−4.1 (3)
C3—C4—C9—C8	179.9 (2)	C11—C10—O3—C7	177.4 (2)
O3—C10—C11—O4	60.5 (3)		

### Hydrogen-bond geometry (Å, °)

**Table:**

*Cg*2 is the centroid of the benzene C4–C9 ring.

**Table:**

*D*—H···*A*	*D*—H	H···*A*	*D*···*A*	*D*—H···*A*
O4—H4···O2^i^	0.82	2.01	2.828 (3)	173
C12—H12C···O1^ii^	0.96	2.54	3.418 (3)	152
C15—H15C···Cg2^iii^	0.96	2.80	3.570 (3)	138

Symmetry codes: (i) *x*, −*y*+3/2, *z*−1/2; (ii) *x*−1/2, *y*, −*z*+1/2; (iii) *x*+1/2, *y*, −*z*+1/2.
